# Effect of laser acupuncture on obesity: study protocol for a randomized controlled trial

**DOI:** 10.1186/s13063-015-0748-4

**Published:** 2015-05-15

**Authors:** Chi-Chuan Tseng, Alan Tseng, Chia-Hao Chang

**Affiliations:** Division of Chinese Medicine, Chang Gung Memorial Hospital, Chiayi, 61363 Taiwan; School of Traditional Chinese Medicine, Chang Gung University, Taoyuan, 33302 Taiwan; Department of Medical Biophysics, University of Toronto, Toronto, M5G 1L7 ON Canada; Chronic Diseases and Health Promotion Research Center, Chang Gung University of Science and Technology, Chiayi Campus, Chiayi, 61363 Taiwan; Department of Nursing, Chang Gung University of Science and Technology, Chiayi Campus, Chiayi, 61363 Taiwan

**Keywords:** Laser acupuncture, Obesity, Body mass index

## Abstract

**Background:**

Obesity-related diseases have a profound economic impact on health care systems. Laser acupuncture has been shown to have beneficial effects on obesity. However, to our knowledge, those trials were either non-randomized, non-blinded or included low-calorie diet control. We have, therefore, designed a patient-assessor-blinded, randomized, sham-controlled crossover trial to investigate the significance of laser acupuncture on obesity.

**Methods/Design:**

104 subjects above 20 years of age with a body mass index (BMI) of over 25 kg/m^2^ will be divided into 2 groups: experimental and control. Each subject will receive the treatment relevant to their group 3 times a week for 8 weeks. After 8 weeks of treatment the subject will enter a 2-week washout period, after which the subjects will switch groups. Measurements will include BMI, body fat percentage, waist-to-hip ratio (WHR), waist circumference, hip circumference, skinfold thickness, thigh circumference, body fat, blood pressure, heart rate, hunger and the 36-item Short-Form Health Survey (SF-36).

**Discussion:**

The results of this study will provide the basis for future large-scale multicenter trials investigating the effects of laser acupuncture on obesity.

**Trial registration:**

ClinicalTrials.gov Identifier: NCT02167308; registration date: 14 June 2014.

**Electronic supplementary material:**

The online version of this article (doi:10.1186/s13063-015-0748-4) contains supplementary material, which is available to authorized users.

## Background

The number of obese people around the world has been increasing rapidly. It has been estimated that over 10% of the world’s adult population are obese [1]. Obesity is associated with increased risk of coronary heart disease, type II diabetes, stroke, osteoarthritis and certain types of cancer. These deleterious conditions are detrimental to individuals’ quality of life, and places emotional and financial burden on the individual, their families and society as a whole [2]. Much more needs to be done to improve treatments for obesity.

Current methods to treat obesity include dietary intervention, lifestyle intervention, drug intervention or bariatric surgery. Among these, acupuncture, in a comprehensive program for weight loss, has been increasing in popularity. However, previous systematic reviews assessing the effects of acupuncture on obesity discovered promising but inconclusive results. The low-quality methodology used in those trials further reduced the strength of those conclusions [3, 4]. In addition, the lack of safety procedures used in traditional manual acupuncture could lead to adverse events such as retroperitoneal abscess, rapid dermal spread of breast cancer, pneumothorax, cardiac tamponade, viral hepatitis and granuloma [5].

In comparison with traditional manual acupuncture, laser acupuncture therapy has several advantages. These advantages include ease of application, precision in dose measurement, painlessness and non-invasiveness. It is a quick and safe therapy with low cost and no risk of infection. As laser acupuncture does not produce local sensation, double-blind randomized controlled studies are also much easier to perform.

Laser acupuncture has been widely used in medical fields for over 30 years. Clinical research on the efficacy of laser acupuncture has also increased [6, 7]. Several studies have shown that laser acupuncture could be an effective treatment of obesity [8, 9]. Low-level laser therapy as a non-invasive approach for body contouring and spot fat reduction has also been reported [10, 11]. However, there are several experimental limitations that have to be considered. For instance, absence of placebo (sham) control, combination with low-calorie diet and lack of sufficient comparative parameters have been questioned [12]. For these reasons, we decided to conduct a patient-assessor-blinded, randomized, sham-controlled crossover pilot trial to investigate the effects of laser acupuncture with Gallium Aluminum Arsenide (GaAlAs) laser irradiation on weight loss, fat reduction, body contouring and quality of life in people suffering from obesity.

## Methods/Design

### Aim of the study

The aim of this study is to assess the effectiveness of laser acupuncture in the treatment of obesity. We will evaluate whether laser acupuncture changes body mass index (BMI), body fat percentage, waist-to-hip ratio (WHR), waist circumference, hip circumference, thigh circumference, body fat, blood pressure, heart rate, hunger and quality of life. All adverse events will also be assessed.

### Study design

This study proposes a patient-assessor-blinded, randomized, sham-controlled crossover trial with a 2-week washout period between each crossover phase. The trials will be conducted at the Chiayi Chang Gung Memorial Hospital. This study will adhere to the recommendations of the Consolidated Standards of Reporting Trials (CONSORT) [13] to allow for greater completeness, transparency and accuracy of reporting. The protocol for this study has been registered in the Clinical Trials register (ClinicalTrials.gov Identifier: NCT02167308).

**Fig. 1:**
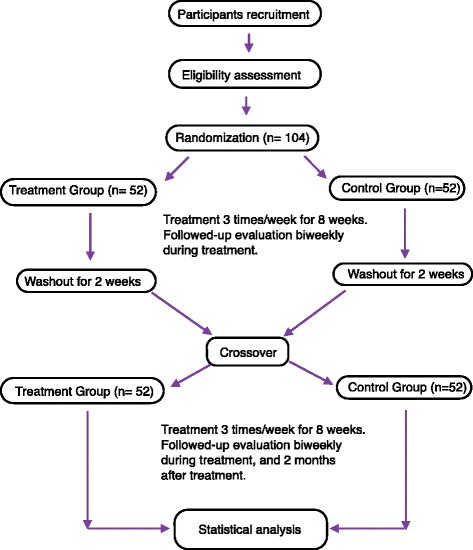
Flowchart of the study design

Subjects who voluntarily sign a consent form will undergo the trial according to the study design. When a subject is determined to be fit for participation based on inclusion and exclusion criteria, the subject will be randomly assigned to 1 of 2 groups in a ratio of 1:1. One group is the true laser experimental group and the other is the sham laser control group. Each group will receive their treatment 3 times a week for 8 weeks. After 8 weeks of treatment and a 2-week washout period, the control group will switch to the experimental group while the experimental group will switch to the control group. The subjects then undergo the treatment of their new group for another 8 weeks (Fig. 1).

Treatment and assessment will be performed independently and the practitioners will not be involved in assessing the outcome of the treatment. The subjects, the outcome assessors and the statistician performing the data analyses will be blinded to the treatment allocation throughout the study.

### Sample size

This study is a pilot study for evaluating the efficacy of actual laser acupuncture compared to sham laser acupuncture, and to evaluate the feasibility of large clinical trials. In the hopes of decreasing the subject drop-out rate, the trial is designed to only last 8 weeks. Assuming a drop-out rate of 10%, the desired sample size for this pilot study is 104 subjects, with 52 in each group. Based on the *a priori* calculation in G*Power 3.1.3 [14] it was determined that a minimum total sample size of 92 would be needed in a repeated measures between factors design to show a significant difference (effect size = 0.25; α = 0.05; 1 – β = 0.8; number of measurements = 8; correlation among repeated measures = 0.2).

### Ethics

This research protocol adheres to the principles of the *Declaration of Helsinki* and has been approved by the institutional review boards of the Chang Gung Memorial Hospital (103-1403A3). Informed consent will be obtained from each participant before any treatment is given. All subjects will have the right to withdraw from the study at any time.

### Inclusion criteria

Individuals are eligible if they are above 20 years of age and have a BMI over 25 kg/m^2^. BMI cutoffs were adopted from a proposed classification of weight by BMI in adult Asians, including the Obese I (BMI: 25 to 29.9 kg/m^2^) and Obese II (BMI ≥ 30 kg/m^2^) categories [15].

### Exclusion criteria

Individuals are not eligible if they have a medical history of cardiovascular disease, diabetes, endocrine abnormalities, renal disease, contagious skin condition, epilepsy, tumors, mental disorders, are pregnant, have photosensitivity reactions to laser treatment, use a pacemaker, have used medications known to affect one’s weight up to 1 month in advance, or are unwilling to comply with the study protocol.

### Randomization and allocation concealment

After initial assessments, subjects will be assigned to 1 of 2 groups with a 1:1 allocation ratio according to a computer-generated randomization list. The group each subject is allocated to will be concealed in sequentially numbered sealed opaque envelopes. The envelopes will only be opened after the subject has completed baseline clinical assessments. Randomization allocation will be concealed from the physician, subjects and evaluators.

### Interventions

#### Laser acupuncture group

Subjects will receive 24 activated laser acupuncture treatments. Treatments will be performed 3 times a week for 8 weeks. Based on the theory of Traditional Chinese Medicine (TCM) and clinical literature on acupuncture therapy for the obese, the acupoints are as follows: ST25 (*Tianshu*), ST36 (*Zusanli*), ST40 (*Fenglong*), ST44 (*Neiting*), LI4 (*Hegu*), LI11 (*Quchi*), SP6 (*Sanyinjiao*) and PC6 (*Neiguan*). An additional file shows this in more detail (see Additional file 1).

The treatment will be applied by a GaAlAs semiconductor diode Laser Phototherapy Device (Model: T-816-3E2-808) developed by Transverse Industries Co., Ltd, Taipei, Taiwan with a wavelength of 808 nm. Maximum power output is 150 mW in continuous wave mode at a power density of 0.417 W/cm^2^. The laser will be applied for 10 seconds to each of the selected acupuncture points with an energy density of 4 J/cm^2^ for each point. This is the recommended treatment dose for low-level laser therapy as documented by the World Association of Laser Therapy [16, 17].

The laser handheld device (Laser Beam Spot Size ≤ 36 mm^2^) will be applied directly and perpendicularly. There will be slight contact with the skin surface to avoid scattering the laser beam. Apart from the light touch of the laser probe when applied to the skin, no sensations were reported when the device was used in the past.

#### Control group

Subjects in the control group will undergo sham laser acupuncture treatment with no laser power output. Acupuncture points, application duration and total number of treatments will be identical to the experimental group. All laser application will be performed by the same physician.

#### Outcome assessment

Before the treatment, clinical assessment parameters will be evaluated by an evaluator. Post-treatment assessments will be evaluated by a different evaluator. The physician who applies the treatment to the subject will not be involved in assessing the parameters. This way, the subject and both evaluators will be blinded to the treatment being performed.

The primary outcome measurement is the BMI change from baseline. BMI will be calculated using weight (kg)/height (m^2^). Secondary outcome measurements include waist circumference, hip circumference, WHR, body fat percentage, hunger and a Short-Form 36-item Health Survey (SF-36). Waist circumference will be measured at the midpoint between the lower margin of the least palpable rib and the top of the iliac crest, using a stretch‐resistant tape. Hip circumference will be measured around the widest portion of the buttocks, with the tape parallel to the floor [18]. Bioelectrical impedance will be used to measure the percentage of body fat.

For hunger, a Visual Analog Scale (VAS) with horizontal lines will be used, graded from 0 mm, representing no hunger, to 100 mm, representing the most extreme hunger imaginable. Subjects will be asked to mark their level of hunger on the VAS. Subjects will be prohibited from accessing their previous VAS records on subsequent sessions. Measurements of appetite as expressed by ratings on the VAS has been shown to be highly reproducible and reliable for appetite research [19]. These clinical assessments will be performed before the trials, once every 2 weeks during the treatment, and 2 months after the last treatment.

SF-36 is a widely used questionnaire for measuring self-reported physical and mental health status. Each subject will answer the SF-36 questionnaire before the trials, immediately after the trials and 2 months after the last treatment. The SF-36 questionnaire consists of 11 questions concerning general health, and can be divided into two aggregate summary measures: the Physical Component Summary and the Mental Component Summary. The SF-36 has become one of the most widely used measures to evaluate health-related quality of life [20].

Other outcome parameters are skinfold thickness, thigh circumference, blood pressure and heart rate. Skinfold thickness will be measured to the nearest millimeter at six sites (triceps, subscapular, suprailiac, abdomen, thigh and calf) using a caliper [21]. All measurements will be taken from the right side of the body. These skinfold thickness measurements will be taken three times, with the average of the 3 values recorded as the representative value. Previous research has indicated that skinfold caliper and computed tomography are similar with respect to the measurement of subcutaneous body fat [22]. The skinfold thickness measurement at different body sites is important because regional adiposity, rather than total excess adiposity, has a greater impact on certain diseases [23].

#### Follow-up

Follow-up observations will be conducted before the trials, once every 2 weeks during the treatment, and 2 months after the last treatment.

### Statistical analysis

Statistical analysis will be performed by using the software program Statistical Package for the Social Sciences (SPSS 15.0, Chicago, IL, USA). Participants’ demographic information and levels of major measured variables were analyzed by descriptive statistics. Means and standard deviations of the clinical indices will be calculated, after which the evaluation between the two groups will be compared using a Student’s *t*-test. The difference of evaluation scores before and after each examination point will be analyzed using paired *t*-tests. The repeated measures analysis of variance (ANOVA) will be used to analyze longitudinal and repeated measured data for the clinical indices across the 8 data collection points (treatments for 16 weeks, followed-up evaluation biweekly). A value of *P* < 0.05 will be regarded as statistically significant for the above statistical analyses.

### Adverse events

Subjects will be asked to report any adverse events they experience. The evaluator will then interview the subjects to confirm the validity of the adverse event. If these events occur during treatment, the physician will immediately stop the procedure and treat the adverse event. The evaluator will then file a report detailing the seriousness of the event, event onset date, relationship between the event and the treatment, possible causes of the event other than the trial itself, and other relevant data. The report will then be sent to the ethics committee. The ethics committee will then decide whether or not to modify the study protocol or remove the subject from the trial.

## Discussion

Obesity is closely linked to many serious health problems and has a profound economic impact on health care systems and society. Current drug treatments for obesity are largely inadequate and there is an enormous unmet clinical need for more safe and efficacious obesity treatments. Laser acupuncture has been shown to have beneficial effects on obesity. However, to our knowledge those trials were either non-randomized, non-blinded, had a limited number of subjects, or included low-calorie diet control. We have, therefore, designed a patient-assessor-blinded, randomized, sham-controlled crossover trial to investigate the effects of laser acupuncture on obesity. A 1-week washout period between treatment sessions was used in a previous randomized crossover study of acupuncture on obesity [24]. We extended this washout period to 2 weeks to further reduce potential carryover effects.

Analysis of skinfold thickness at different body sites, feeling of hunger and quality of life will also be performed. To our knowledge, these secondary outcomes have not yet been measured in similar studies investigating the effects of laser acupuncture on obesity. Through this study we will be able to determine whether or not laser acupuncture can alter regional adiposity, body contour and sense of well-being. We hypothesize that appetite suppression and circumferential reduction through laser acupuncture can help people achieve better self-control in diet and exercise. If our hypothesis is correct, laser acupuncture may play a role in encouraging subjects to adhere to changes in behavioral lifestyle.

Previous research has suggested that gender moderates the psychological and behavioral variables on weight loss treatment [25]. However, the relevance of gender on weight loss differences with respect to laser acupuncture remains unexplored. Unlike previous studies, we will equalize the sample sizes of the two genders to help elucidate this substantial variability in outcomes. This trial seeks to provide clinical data on the effect of laser acupuncture on BMI, body fat, WHR, body contour, appetite and quality of life in the obese. Results of this trial will help clarify the value of laser acupuncture as a treatment option for individuals suffering from obesity.

## Trial status

This trial is currently recruiting participants.

## Additional file

Additional file 1:
**Anatomical locations of the acupuncture points.**

## Abbreviations

ANOVA: analysis of variance
BMI: body mass index
CONSORT: Consolidated Standards of Reporting Trials
GaAlAs: Gallium Aluminum Arsenide
SF-36: Short-Form 36-item Health Survey
TCM: Traditional Chinese Medicine
VAS: Visual Analog Scale
WHR: waist-to-hip ratio
