# Supplementary material for: Effect of laser acupuncture on obesity: study protocol for a randomized controlled trial
Source: Trials. 2015 May 15;16:217. doi: 10.1186/s13063-015-0748-4 (PMC4440285; doi:10.1186/s13063-015-0748-4)
Supplement: Additional file 1: — Anatomical locations of the acupuncture points. [file 13063_2015_748_MOESM1_ESM.pdf]

Additional file 1. Anatomical locations of the acupuncture points

Acupuncture point	Anatomical location	Function according to TCM
ST 25	Middle of the abdomen, 2 cun lateral to the umbilicus.	Regulates spleen, stomach, and intestines.
ST 36	3 cun below patella, 1 cun lateral of the tibial rim.	Strengthens spleen and stomach.
ST 40	At the midpoint of a line between ST35 at the lateral patella and the lateral malleolus.	Transforms phlegm and dampness.
ST 44	Proximal to the web margin between the 2nd and 3rd metatarsal toes, at the junction of the red and white skin.	Clears heat from stomach, resolves damp heat.
LI 4	On the dorsum of the hand, between the 1st and 2nd metacarpal bones, in the middle of the 2nd metacarpal bone on the radial side.	Expels wind and releases the exterior, tonifies qi and strengthens immunity
LI 11	With the elbow flexed, the point is on the lateral end of the transverse cubital crease.	Clears heat, cools blood, resolves dampness, expels exterior wind.
SP 6	3 cun above the medial malleolus, dorsal tibial rim.	Strengthens spleen and stomach, resolves dampness.
PC 6	2 cun above the transverse crease of the wrist, between the tendons of m. palmaris longus and m. flexor carpi radialis.	Regulates heart qi, calms the shen, harmonizes stomach.
